# Protocol for a randomized controlled trial testing the impact of feedback on familial risk of chronic diseases on family-level intentions to participate in preventive lifestyle behaviors

**DOI:** 10.1186/s12889-016-3623-7

**Published:** 2016-09-13

**Authors:** Carlene J. Wilson, Kayla de la Haye, John Coveney, Donna L. Hughes, Amanda Hutchinson, Caroline Miller, Ivanka Prichard, Paul Ward, Laura M. Koehly

**Affiliations:** 1 Flinders Centre for Innovation in Cancer, Flinders University of South Australia & Cancer Council South Australia, Adelaide, Australia; 2 Institute for Health Promotion & Disease Prevention Research, University of Southern California, Los Angeles, USA; 3 School of Health Sciences, Flinders University of South Australia, Adelaide, Australia; 4 School of Psychology, Social Work and Social Policy, University of South Australia, Adelaide, Australia; 5 South Australian Health and Medical Research Institute, Adelaide, Australia; 6 Social and Behavioral Research Branch, National Human Genome Research Institute, Bethesda, USA

**Keywords:** Intergeneration transmission, Eating behavior, Health behavior, Family health history, Randomised clinical trials, Culture

## Abstract

**Background:**

Common disease risk clusters in families due to shared genetics, exposure to environmental risk factors, and because many health behaviours are established and maintained in family environments. This randomised controlled trial will test whether the provision of a family health history (FHH) risk assessment tool increases intentions and engagement in health behaviors. Message distribution and collective behavior change within family networks will be mapped using social network analysis. The relative intervention impact will be compared between families from different ethnic backgrounds.

**Methods:**

One hundred and fifty mothers (50 Anglo-Australian, 50 Italian-Australian, 50 Vietnamese-Australian) will be recruited, with four or more other family members across three generations, including a child (aged 10–18 years). Each family is randomly assigned to intervention or control. At baseline and 6-month follow-up, all participants complete surveys to assess dietary and physical activity intentions and behaviors, attitudes towards food, and perceived disease risk. Intervention families receive a visual pedigree detailing their FHH of diabetes, heart disease, breast and bowel cancer, a health education workbook to ascertain members’ disease risk (i.e. average or above average risk), and screening and primary prevention recommendations. After completion of follow-up assessments, controls will receive their pedigree and workbook. The primary hypothesis is that attitudes and lifestyle behaviors will improve more within families exposed to FHH feedback, although the extent of this improvement may vary between families from different ethnic backgrounds. Additionally, the extent of improvement in the treatment group will be moderated by the level of family disease risk, with above-average risk leading to greater improvement. A secondary aim will explore different family members’ roles in message distribution and collective responses to risk using social network approaches and to compare network functioning between families with different ethnic backgrounds.

**Discussion:**

Results will guide future health promotion programs aimed at improving lifestyle factors. This research will assess whether FHH can motivate families to adopt family-level strategies to support health promoting behaviors. Secondary analyses aim to identify change agents within the family who are particularly effective in shifting normative behaviors.

**Trial registration:**

Australian New Zealand Clinical Trials Registry ACTRN12613001033730. Retrospectively registered: 17 September, 2013.

## Background

Lifestyle-related diseases including heart disease, stroke, diabetes, obesity, metabolic syndrome, chronic obstructive pulmonary disease, and some types of cancer are endemic in many developed countries. These diseases share some or all of four behavioral risk factors; an unhealthy diet, physical inactivity, alcohol consumption, and smoking. The United Nations describes these as “Western diseases” or “diseases of affluence” [1]. The health burden in Australia mirrors this finding, with data suggesting that one third of the nation’s disease burden is linked to poor lifestyle choices [2]. These diseases are etiologically complex. They arise from genetic factors and environments that promote the normalisation of unhealthy food consumption through exploiting human vulnerabilities, be these biological, psychological, social or economic [3–5].

To date, health interventions and policy to reduce lifestyle-related disease have largely adopted behavior change strategies that focus on the individual. However, focusing on families rather than individuals may be a more effective strategy for disease prevention because family members share genetic risk, they are typically exposed to similar environmental risk factors, and health-compromising behaviors tend to cluster within families. Indeed, there is evidence that both food choice [6] and eating behavior [7] show strong consistency in families, even across generations. This highlights dietary practices as an avenue for family-based interventions designed to modify disease risk [8]. Similarly, physical activity is also shown to aggregate within families again highlighting the family context as potentially important for behavior change [9, 10]. In addition, effective use of family health histories (FHHs) as part of family-based disease prevention efforts is a promising mechanism for motivating collective health behavior change. Family health histories capture the complex interactions between shared genetic, lifestyle and environmental factors, and so are ideally suited to informing family-based disease prevention [11]. The potential utility and acceptability of FHH are supported by data from the US Center for Disease Control which, in 2004, reported that the vast majority of the US population (more than 96 %) considered that knowledge of family health history was important to their personal health [12]. This study is concerned with assessing the impact of the provision of families’ personalised FHHs on intentions to modify diet, physical activity and other lifestyle behaviours, and subsequent behaviour, in families with different ethnic backgrounds.

### Family health history assessments to promote disease risk-reducing behaviors

The U.S. Surgeon General and Centers for Disease Control and Prevention (CDC) have instigated FHH initiatives to provide web-based tools designed to encourage individuals to map their family’s history of heritable chronic diseases [13]. To accelerate the use and impact of FHH tools, initiatives have sought to promote behaviors to reduce disease risk directly. These FHH assessment programs typically engage individuals and involve the analysis of information on family morbidity from heritable diseases; feedback on individuals’ disease risk based on FHH trees; and behavioral strategies for risk modification [13–16] “Families SHARE” is one such tool with demonstrated efficacy [17].

Research suggests that individuals provided with a FHH-based risk assessment can make significant improvements to their lifestyle, particularly when provided with individually tailored behavior change information aimed at modifying their disease risk [18]. For example, Sato Ashida, Wilkinson and Koehly [19] completed a longitudinal FHH intervention with Mexican American families and found that participants who received messages of family disease risk (based on a FHH) reported a higher motivation to increase their intake of fruit and vegetables. Further, parents may be particularly motivated to improve their own and their child’s health in response to family risk information. Indeed, FHH-based risk information spurred parent-child encouragement of physical activity, which in turn was associated with parents and children co-engaging in physical activity in the study of Mexican American families [20]. Research by this team has also found that family members with disease experience play a vital role in disseminating information and encouraging or advocating health behaviors in the family, highlighting the potential to harness existing relational processes of communication or influence to improve dietary, physical activity, and other health behaviors within family systems [21].

*Families SHARE*, which will be used in this project, is a FHH tool designed to facilitate understanding of the role of FHH in disease risk by the public [17]. The Families SHARE workbook consists of 1) a personalized pedigree representing family morbidity from specific diseases (including diabetes, heart disease, and cancer), 2) an algorithm for computing a risk assessment based on the family pedigree, and 3) behavioral strategies for risk modification. A recent evaluation of the workbook found that users reported significant increases in intentions to improve dietary behaviour following use of the workbook, suggesting that Families SHARE may be successful in shifting dietary behaviors within the home. In addition, users who were mothers of young children, were able to apply the algorithm to assess their own and other family members’ risk. Feedback indicated a desired focus on their child’s risk, with the suggestion that such a focus would improve parent motivation to shift towards more healthful norms within the home. Taken together, these results suggest that Families SHARE may be a particularly useful tool within a family-based intervention that leverages parent protection motivations geared towards children.

As such, provision of FHH information within a family-centered feedback process may initiate communal coping. A communal coping response involves family members communicating about a health threat (i.e., family risk of disease), developing a shared appraisal of that threat, and engaging in cooperative action to address the threat [22–24]. This would result in an activation and shift in communication, influence, and support among family members, which can heighten awareness of family disease risk and facilitate the adoption of risk reducing behaviors. Despite the promise of FHH initiatives for motivating risk reducing health behaviors within families, there remain two critical gaps in these translation efforts: a lack of research that (1) adapts and evaluates these tools for diverse populations, who have varied family structures and family social dynamics; and (2) explicitly accounts for family systems and their role in the success of FHH interventions. These gaps can be addressed through a cluster randomised controlled trial that provides FHH-based risk information, such as Families SHARE, to diverse, multigenerational families. The extent to which families are impacted is likely to be dependent on actual risk level, habitual behaviors, and cultural traditions with regard to food and lifestyle. Further, research that maps the interpersonal mechanisms that comprise communal coping as potential process variables is limited. Thus, capturing the network of communication and influence relationships among family members will allow us to understand how diverse family structures and systems moderate or mediate intervention effects.

### Transmission of disease risk and related behaviors: the role of family and culture

To a large extent, family health environments are shaped by parents’ beliefs, attitudes, and behaviors, education, and ethnic/cultural backgrounds and norms [25]. Together these factors may create family environments that are more or less promoting of disease risk. Interventions that increase motivation within the family to adopt and maintain healthy behaviors may, in the long term, reduce the prevalence of lifestyle related diseases.

The intergenerational and social ‘transmission’ of lifestyle diseases such as obesity [26], and associated health-risk behaviors emphasize the importance of social processes within families - i.e., family social networks dynamics - and family culture, in reinforcing or changing health behaviors. For example, Christakis and Fowler [26] highlighted the link between interpersonal relationships (i.e., “social ties”) and weight in a large community social network (N = 12,067), with evidence of similarities in weight status among siblings, spouses and friends over time. Subsequent research suggests that similarities in weight status (and potentially other related health outcomes) among socially connected individuals are in part due to social networks directly influencing individual health behaviors (including eating [27–29] and physical activity [30, 31]), and indirectly influencing these behaviours by shaping weight norms [32, 33]. Traditional social psychological research has established that social influence on eating and activity can occur through a variety of interpersonal and group processes including modelling behavior, normative influence, ‘mindless imitation’ and social facilitation.

The impact of social influence on health behaviors is likely to be especially prominent in families, particularly where children still live at home with their parents. There is a strong interplay between parents’ health behaviors and associated health outcomes (i.e., overweight), and children’s health trajectories [34, 35]. For example, the links in dietary behaviors among family members are especially strong. Parents appear to play a particularly important role in the establishment of dietary behavior in children that endures into adulthood [36]. Prichard, Hodder, Hutchinson and Wilson [37] confirmed a strong association between adult daughters’ dietary intake of energy-dense snack foods and vegetables and their perceptions of their mothers’ intake of these same foods with observed correlations of .81 and .52, respectively. These correlations are not entirely due to the number of meals shared, particularly with regards to daughters’ intake of energy dense foods. Data collected on eating behaviors (i.e., engagement in Restrained Eating, External Eating and Emotional Eating) also highlighted strong mother-daughter similarity with correlations between mother and daughter of .33, .52 and .62 respectively for the three behaviors.

There is also evidence of a link between the broader family environment and eating behaviors. Energy dense food consumption and healthy food choices are positively correlated among family members, both across and within generations. For example, fat intake has been found to correlate between parents, mother and child, and father and child [6]. However, family influence on food is also complex, and has been found to differ based on the closeness of the family tie, as well as by family role or position (e.g., child, parent or grandparent), and may also differ across ethnic groups [38].

Ethnicity and associated cultural practices also have an important influence on eating behaviors and health outcomes for families. In Australia, patterns of lifestyle diseases such as obesity in children can be linked to the sub-continental origins of the parents [39]. Italian-Australians have been found to have a higher incidence of overweight than the population, whereas the rate amongst Vietnamese-Australians is lower [40]. Moreover, cancers linked to dietary and other lifestyle choices occur at a significantly higher incidence among some immigrant groups than is found in their country of origin, suggesting that the adoption of Australian normative behavior may be causally associated with this outcome [41]. Identifying how the transmission of eating practices differs across these ethnic groups may allow us to identify influence processes that are protective of, or promote lifestyle-related disease.

Bidirectional, intergenerational influences on eating habits have been documented in families of diverse ethnic backgrounds [25], with children potentially playing an important role in the adoption of ‘new’ behaviors [20, 42]. It is also clear that other family members fulfil powerful roles: mothers in virtually all cultures play a critical role through primacy in meal preparation, whereas grandparents have been found to significantly influence their grandchildren’s diets in some cultures [4]. Families of different social and ethnic backgrounds may differ in their interpretation of food and health information [43], which is likely to impact the salience and meaning of diet in the family. How this impacts understanding and communication of food and health issues in families will be examined in the proposed study.

### The proposed study: using families SHARE to promote healthy eating and other disease risk-reducing behaviors in families with diverse ethnic backgrounds

The literature reviewed above suggests that an intervention providing families with a FHH-based risk assessment focused on lifestyle linked-diseases in conjunction with recommendations towards risk-reducing health behaviors may serve as a motivation for behavior change within families. The Families SHARE workbook provides both a risk algorithm that can be applied to multiple family members as well as lifestyle and screening recommendations focused on disease prevention and early detection. It is hypothesized that provision of the Families SHARE workbook will activate family social network processes which in turn will influence and support the adoption of the promoted health behaviors.

The extent to which families are impacted by such a tool is likely to depend on actual disease risk level, habitual behaviors, communal responses within families, and food and meal traditions associated with ethnic background. Psychosocial variables likely to influence responsiveness to information about familial disease risk and responses on these variables, in turn, may be moderated by age, gender, education and ethnic background. These include differences in social and cultural capital [44, 45], optimism [46] and health locus of control [47]. Each of these variables has a demonstrated relationship to participation in health-related behaviors, self-reported health, or morbidity and mortality and may serve to impact responsiveness to interventions designed to provide motivation to improve lifestyle choices.

Notwithstanding the potential influence of individual difference variables, the proposed study will focus on the family cluster as the primary unit of observation. The primary aim is to discern the impact of inter and intragenerational influence on family food intentions and choices following exposure to a self-generated pedigree of family disease risk. Given past research, which has highlighted ethnic group differences in food intake and meal-time behaviour, this factor will be considered as a potential possible moderator of the impact of exposure to Families SHARE.

The remaining text describes the protocol for a randomised controlled trial of Families SHARE among multigenerational families of diverse ethnic backgrounds in Australia.

## Methods/design

### Study aims

The main aim of the study is to test whether the provision of a FHH risk assessment workbook, Families SHARE, for families to use to identify their familial chronic disease risk and as an algorithm for them to identify the individual risk of any specific family member impacts intention to change diet and actual diet. The potential moderating impact of ethnic background will be considered. A secondary aim is to examine how the intervention alters relationships within family social networks, including the diffusion of messages about disease risk, and the activation of networks that influence and support behaviors, to understand the role family systems play in moderating behaviour change and to understand if the manner in which information provided by the FHH influences diffusion within the networks.

### Study design

**Fig. 1:**
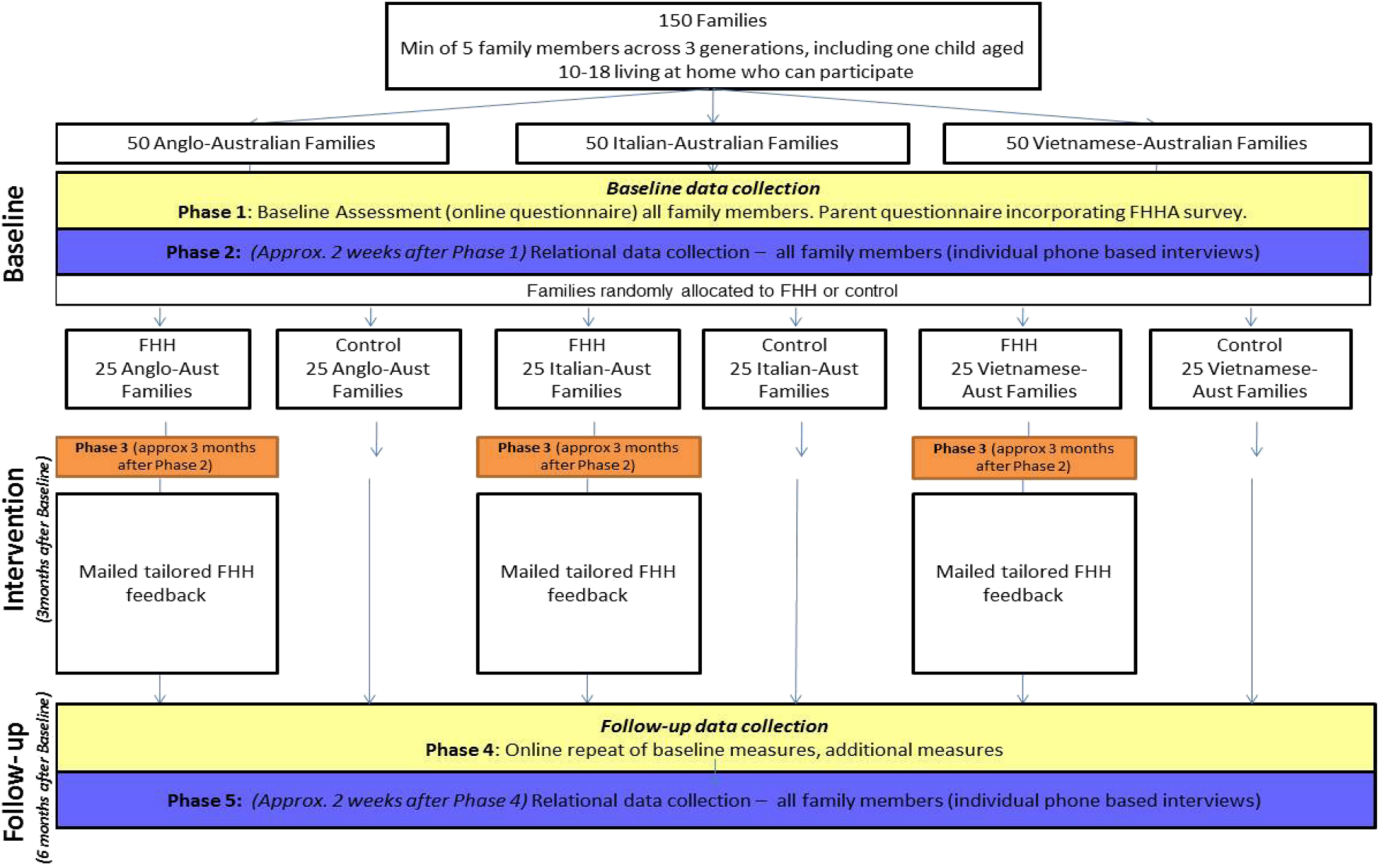
Flow diagram of participant progress through study

This study is a cluster, randomised controlled trial where mothers from Anglo-Australian, Italian-Australian and Vietnamese-Australian ethnic background will be randomly assigned to an intervention (provision of Families SHARE) or control condition (received nothing during the study). The study is longitudinal, with six months elapsing between the completion of baseline and the commencement of the follow-up. For those in the experimental condition, the Families SHARE workbook along with personalized pedigree (i.e., intervention) will be mailed to participants three months after baseline assessment, at the midpoint of the study. The trial flowchart with time-frames is shown in Fig. 1.

### Ethical considerations

Full ethics approval has been obtained from the Social and Behavioural Research Ethics Committee at Flinders University of South Australia.

### Recruitment of eligible participants

One hundred and fifty mothers (“index mothers”) representing three ethnic groups, Anglo-Australian, Italian-Australian, and Vietnamese-Australian, will be targeted for the first stage of recruitment, based on the following selection criteria: having at least one child aged 10–18 living at home who could participate in the study; and having a minimum of four additional family members (including their child), that span three generations who are living in Australia and who would be eligible to participate in the study. Recruitment will occur by means of advertisement in ethnically targeted areas of the city, via assistance from key community members and organizations, and from invitation letters sent home with children at targeted schools. Interested potential participants will contact the research team who will then ascertain the family’s eligibility to participate by conducting a short eligibility survey via telephone. Interested participants will also be asked to identify their ethnicity and the country of birth of their parents. Families will be categorized into Anglo-Australian, Italian-Australian or Vietnamese-Australian cultural groups based on the birth country of the index participant and their parents, as well as the ethnicity that the family reports to identify with.

Once eligible index mothers and their families are identified, children will be recruited into the study (based on parental consent), and multiple attempts will be made to enrol at least three additional family members based on the index mother’s referral. Families who do not identify with the cultural eligibility criteria or who only have interested participants spanning two generations or less than five members will be deemed ineligible.

All recruited families will receive reimbursement for their time and inconvenience with $50 given to each family after return of all baseline survey data and a further $50 per family after receipt of endpoint survey data. The $50 will be presented each time in the form of five $10 shopping vouchers, one for each participating family member.

### Randomisation

Within each ethnic group, each family will be randomised to either the intervention or control condition upon entering the study. A simple randomisation table will be created pre-trial using Microsoft Excel. Each family that enters the study will be allocated the next available randomised condition provided on the list.

#### Baseline survey

At baseline, all participants (i.e., all family members nested in the 150 families) will complete a survey to assess lifestyle behaviors, including diet and physical activity, attitudes towards food and perceived disease risk. This survey will be available in either a paper or online format and takes no longer than 45 min to complete. There are two baseline forms of the questionnaire, one for adults and one for children. The adult version is identical across ethnic groups, with the exception of an acculturation questionnaire which appears in the Italian and Vietnamese versions. As part of their baseline survey all index mothers and fathers will be invited to complete a FHH, which involves indicating disease history for heart disease, diabetes, breast and colorectal cancer for the mothers’ and fathers’ first and second degree relatives. Measures included in the parent and child versions of the baseline are described in Table 1.

**Table 1:** Summary of measures included and point of data collection

Families, food and eating survey	Baseline	Endpoint	Adult	Child
Section 1: Cultural identity and background (7 items)				
Country of origin and parents’ origin, language spoken at home.	X		X	X
Section 2: Lifestyle (current behavior determined from previous week)^1^ (13 items)				
Smoking status	X	X	X	
Vigorous Physical Activity:				
• Days per week	X	X	X	X
• Hours/Minutes per day	X	X	X	X
Moderate Physical Activity:				
• Days per week	X	X	X	X
• Hours/Minutes per day	X	X	X	X
Sedentary Behavior:				
• Hours/Minutes per day on a weekday	X	X	X	X
• Hours/Minutes per day on the weekend	X	X	X	X
• Sedentary activities (Children only) – hours per day on a weekday	X	X		X
• Sedentary activities (Children only) – hours per day on a weekend	X	X		X
Family physical activity				
• Days per week	X	X	X	X
• Hours/Minutes per day	X	X	X	X
Serves of fruit per day averaged over previous week	X	X	X	X
Serves of vegetables per day averaged over previous week	X	X	X	X
Number of visits to franchised fast food restaurants in the previous week	X	X	X	X
Serves of high fat and/or high sugar “snacks” per day averaged over previous week	X	X	X	X
Alcohol consumption				
• Days on which alcohol consumed in past week	X	X	X	
• Average number of standard drinks on drinking days	X	X	X	
Section 3: Intended Lifestyle Changes (Minimum 1 item, Maximum 6 items)^2^				
Considering Lifestyle change (yes or no)	X	X	X	X
Areas: Fruit and Vegetable consumption; Physical Activity;	X	X	X	X
Additional areas: Fibre consumption; Alcohol consumption; Smoking	X	X	X	
If yes, identified each area of change and confidence in capacity to change (Self efficacy for Behavior Change) on a 7 point rating scale where 1 is Not at all Confident, 4 is Moderately Confident and 7 is Very Confident.	X	X	X	X
Indicate any areas where lifestyle changes have been made in the last 6 months		X	X	X
Section 4: Food Attitudes (21 items)# (some excluded for children; 16 items)				
Food Life Questionnaire Short Form [53] rated on a 7 point rating scale where 1 is Strongly Disagree and 7 is Strongly Agree	X	X	X	X
Section 5: Disease Risk (10 items)				
Likelihood of talking to doctor about chronic diseases risk in the next 6 months	X	X	X	
Likelihood of talking to family about chronic diseases risk in the next 6 months	X	X	X	
Ratings of perceived lifetime risk for colorectal cancer, breast cancer, heart disease and diabetes	X	X	X	
All rated on a 7 point rating scale where 1 is Not at all Likely and 7 is Extremely Likely. (Don’t Know and Not Applicable provided for each specific disease ratings)				
Perceived contribution of lifestyle factors to disease risk (includes eating habits, alcohol consumption, physical inactivity, genetic factors)	X	X	X	
All rated on a 7 point rating scale where 1 is Not at All and 7 is A Great Deal				
Section 6: Cultural Identity (Italian and Vietnamese participants only)				
Stephenson Multigroup Acculturation Scale (32 items) [54]	X		X	
Ratings of extent of agreement with a series of “I” statements				
All rated on a 4 point rating scale where 1 is False and 4 is True. Not Applicable also provided.				
Section 6 (or 7): Questions about your *own* health history (1 item; 4 diseases)				
Categorical response for heart disease, diabetes, colorectal cancer and breast cancer (includes Don’t Know and Age at diagnosis)	X		X	
Section 7 (or 8): Questions about your *family’s* health history (9 items) (used to compile FHHA; adapted from CDC online Family Healthware Tool)				
Categorical response for heart disease, diabetes, colorectal cancer and breast cancer (includes Don’t Know and Age at diagnosis)	X		X	
Section 8 (or 9): Demographics (12 items)				
Age, gender, height, weight, number of people living in the home, number of children (adults) or siblings (children), adults only: marital status, income (Endpoint only), education (Endpoint only), health insurance (categorical)	X	X	X	X
Social and Cultural Capital (6 items/domains)				
Newspaper most often read (categorical)	X		X	
Television station watched most often (categorical)	X		X	X
Shops from which food is purchased and frequency, choice of 4 including “Other”	X		X	
All rated on a 5 point rating scale where 1 is Never and 5 is all the time				
Recreational activities, choice of 6	X		X	
All rated on a 5 point rating scale where 1 is Never and 5 is daily				
Frequency of direct contact with three groups of people; friend, workmates and neighbors	X		X	
All rated on a 6 point rating scale where 1 is Almost every day and 6 is Don’t have any				
Membership in any of 4 different organizations; religious, recreational, cultural or educational, and “other” community-based (categorical)	X		X	
Endpoint – Additional Measures				
Section 4: Beliefs and Attitudes to Life (28 items)				
Multidimensional Health Locus of Control Scale (18 items) [55]		X	X	
Life Orientation Test – Revised (10 items) [56]		X	X	
All rated on a 5 point rating scale where 1 is Strongly Disagree and 5 is Strongly Agree				
Intervention group only – evaluation of the extent to which Families SHARE information was shared amongst family members		X	X	
Section 5: Disease Risk (15 items)				
Conversations about chronic diseases in the past 6 months with doctor, family member, friends, others	X	X	X	
Ratings of perceived lifetime risk for colorectal cancer, breast cancer, heart disease and diabetes *of child*	X	X	X	
Rated on a 7 point rating scale where 1 is Not at all Likely and 7 is Extremely Likely.				
(Don’t Know and Not Applicable provided for each specific disease ratings)				
Participation in screening for each of the 4 chronic diseases		X	X	
Section 6: Your Family				
Box providing scope for enumeration of people who are considered as family members		X	X	X
Section 7: Family Health History Evaluation INTERVENTION GROUP ONLY (3items; all categorical responding)				
Ability to assess disease risk		X	X	
Sharing of FHHA information		X	X	
Was the FHHA updated after receipt?		X	X	

^1^Section 1 in Endpoint Survey

^2^Section 2 in Endpoint Survey

#### Family social network survey

Approximately 2 weeks after return of the final family member’s baseline survey, each family member will be contacted to arrange a phone interview to gather information on their family social network. A personal, or “egocentric”, approach to data collection will be adopted [48], whereby participants are asked to name the people in their family and answer questions about the types of relationships they have with each family member. Participants will first be asked to enumerate a list of individuals they consider to be “family members”, which can include biological kin, non-biological kin, and “social kin”. Then they are asked to consider the previous 3 months and to identify which of these family members they shared the following specific types of relationships with: with whom they talked to about health and family risk of disease; who encouraged them to eat healthily or unhealthily or made eating specific healthy or unhealthy foods difficult; with whom they exercised, who encouraged them to exercise or who made exercising difficult to engage in; and family members with whom they watched television. Adult participants will also be asked to consider the same 3 month period and identify with whom they consumed alcohol, who encouraged or discouraged consumption, and who made it difficult to consume. The adults will be asked the same questions about smoking (see Table 1).

#### Family health history intervention

Three months after an intervention family has provided the last baseline survey they will receive a health education booklet, entitled ‘Families SHARE’. This will contain a visual family pedigree of the previously identified first and second degree relatives of the index parents with disease markers highlighted for heart disease, diabetes, breast and colorectal cancer. The ‘Families SHARE’ booklet contains a clear description of a family pedigree with guidance to help participants interpret their own and other family members’ risk for disease using the pedigree. In addition, the booklet contains information about genetic and environmental risk for disease along with behavioral and screening recommendations.

The control group of families will receive nothing between baseline and follow-up, and will receive their ‘Families SHARE’ booklet containing their family’s health history upon completion of the study.

#### Endpoint survey

All participating families will be re-contacted 6 months following the completion of baseline for follow-up assessment. This endpoint survey is similar in format to baseline. It will assess eating and exercise behaviors, attitudes towards food and perceived disease risk, health locus of control, optimism, as well as assess intentions to increase healthy behaviors in the future and tap any purposeful changes made or screening activities undertaken in the past 6 months. In addition, adults in the intervention group will be asked questions designed to evaluate the extent to which information from the Families SHARE workbook was shared amongst family members. All children in the trial will complete the same children’s version of the questionnaire (see Table 1).

#### Follow-up assessment of family social network survey

Approximately 2 weeks after the collection of the endpoint survey data, family members will be recontacted and the social network data will be recollected via individual phone interview (see Table 1).

### Outcome measures

The primary outcome tested will be the changes in intentions to improve food choices following exposure the FHH the main hypothesis is that exposure to the FHH will improve intended dietary behaviour with the extent of improvement dependent about the nature of risk within the family (i.e., above average v average). Secondary analyses will focus on exploration of the communication, social influence, and support networks within families at baseline, and how these differ between cultural groups and are related to health behaviors. Changes in the patterns or ‘structure’ of these social networks after exposure to the Families SHARE intervention will be compared to changes in the control families. For example, we will test if exposure to Families SHARE activates a communal coping response, evidenced by an increase in density of communication ties and encouragement ties among family members, and if this moderates health behaviour outcomes.

#### Sample size

Power analyses were conducted to estimate the number of families needed to evaluate the impact of the intervention on intentions to change diet. The sample size estimates consider potential clustering of the outcome variable in families, and is based on a multilevel generalized linear model assessing the effect of intervention (intervention vs. control; level 2 variable) on behavioural intentions at follow-up. Covariates considered within the fitted model include the baseline intentions (level 1 variable) and ethnic group (level 2 variable). Since the intervention component is a level 2 variable, the power calculations optimize the macro-level units [49]. Based on a medium effect size (Cohen’s f = .25, ω^2^ = .06), a Type I error rate of .05, we would need, conservatively, 132 families to achieve a power equal to .80. As the time between baseline and the final assessment is short, we expect retention rates to be relatively high and so an initial sample of 150 families will be recruited.

### Statistical analyses

#### Primary outcomes

The primary outcome is change on the diet-related intentions of each individual in the study, according to group assignment (Treatment (FHH) versus Control), controlling for ethnic background (Anglo, Italian, and Vietnamese). The primary outcomes are; intentions to change diet, specifically, fruit and vegetable and fibre consumption. Secondary outcomes include intentions to change physical activity, alcohol consumption and smoking. These initial analyses will consider differential effectiveness of the intervention according to extent of family risk for chronic disease and the ethnic background of the families.

While the time period between assessments is short, which will limit potential behavioural changes over the 6-month period, levels of consumption and participation in ancillary health-linked lifestyle choices will also be assessed and compared between groups and over time. In addition, we will establish the extent to which change is moderated within the intervention group by the nature of the feedback (average v above average risk) and the potential moderating or mediating effects of cultural and social capital, health locus of control, optimism in the full sample, and acculturation within the minority groups.

A multilevel modelling approach, which accounts for participants being nested in families, will be used to look at the effect of the intervention, and ethnicity, on intentions to change behaviors post intervention. We hypothesize a main effect for the intervention condition so that intentions and behavior become more consistent with feedback recommendations following exposure to the FHH assessment than does that of the control group. Comparison between ethnic groups will test for efficacy differences.

#### Secondary outcomes

Building on the study of Feunekes et al. [6], we will first explore the extent to which diet and eating behaviors correlate amongst different types of family ties at baseline (e.g., parent-child, spouse, etc). Social network analyses will then be undertaken to summarize the characteristics of family social networks at baseline and follow up, which will allow us to test if there is differential change in the structure of these networks across study conditions, and across ethnic groups. For example, we expect the density of communication ties (i.e., proportion of actual relative to possible ties) and density of encouragement ties among family members receiving the intervention to increase, driven by a communal coping response. Relational structures such as *mutual* influence and encouragement will also be analysed, to understand their role in promoting change in the main dietary outcome measures, and to determine if these processes differ across intervention and control conditions, and across ethnic groups. Social network analysis will also be used to describe the roles of various family members in these health communication and support networks, to identify family members playing important roles in initiating or maintaining the targeted health behaviors across three generations, and family members who play key roles in facilitating or hindering behaviour change.

### Time plan

Participant recruitment will be ‘rolling’, as families enter the study they will commence baseline and their own 6-month time-frame.

## Discussion

To examine intergenerational transmission of health-related behaviors in families with different ethnic backgrounds, and to assess the impact of FHH-based risk assessment across generations in an appropriately controlled way, it is necessary to apply very strict inclusion criteria. These include the need to have members from at least three generations available to participate; enrolling participants who are prepared to provide information about their family’s health history; potentially collecting data from “blended families” and requiring at least a moderate level of English language skill from most participants.

Enrolling families using these criteria is likely to require significant time. Moreover, the proposal to identify families through the mother may create further difficulties, particularly where English language is poor and the mother is at home. There is little guidance available in the research literature on best practice for recruitment of complex sampling frames that involves people from multiple ethnic backgrounds and age groups. We will therefore use the experience garnered in the proposed study to provide a case study report of the problems encountered and any variations required to the approaches described earlier.

The possibility of attrition associated with the longitudinal design and complex requirements will need to be closely managed. We hope to minimise this by the design and distribution of a regular newsletter highlighting progress in the study and the importance of participation and including items that might be of interest to the families (e.g., recipes and local events) [50].

The issues of research translation and generalizability will not be adequately addressed within the context of the current study design. Our focus is on efficacy rather than effectiveness and managing threats to internal rather than external validity. Assuming efficacy can be established; further work will be required to address the process by which exposure to family history of disease might best be achieved. Additionally, the receptivity of other groups in the community to this information cannot be assumed. Anglo, Italian and Vietnamese Australians were selected for the current study because they are among the larger groups in South Australia, improving the probability of recruiting the required numbers [51]. Moreover, the two non-English speaking groups represent different waves of migration to Australia [52], different incidences of overweight and different traditional eating habits [40, 41]. If the intervention is found to be efficacious in motivating intentions to change lifestyle behaviors in families, it will be necessary to evaluate its use and effectiveness in the wider population, across groups with different ethnic and other background characteristics.

## Abbreviation

FHH: Family health history
